# Supplementary material for: Concurrent improvements in maize yield and drought resistance through breeding advances in the U.S.Corn Belt
Source: Nat Commun. 2025 Oct 23;16:9389. doi: 10.1038/s41467-025-64454-3 (PMC12549856; doi:10.1038/s41467-025-64454-3)
Supplement: Supplementary file 1 — Supplementary Information [file 41467_2025_64454_MOESM1_ESM.pdf]

Supplementary Information

Concurrent improvements in maize yield and drought resistance through breeding advances in the U.S. Corn Belt

Haidong Zhao[1], Jesse B. Tack[2], Gerard J. Kluitenberg[1], M. B. Kirkham[1], Gretchen F. Sassenrath[3], Lina Zhang[1], Nenghan Wan[1], Zhijuan Liu[4], Jin Zhao[4], Amanda Ashworth[5], Prasanna H. Gowda[6] & Xiaomao Lin[1*]

[1]Department of Agronomy, Kansas State University, 2004 Throckmorton Plant Sciences Center, Manhattan, KS 66506, USA

[2]Department of Agricultural Economics, Kansas State University, Manhattan, KS 66506, USA

[3]Kansas State University Southeast Research and Extension Center, 25092 Ness Road, Parsons, KS 67357, USA

[4]College of Resources and Environmental Sciences, China Agricultural University, No. 2 Yuanmingyuan West Rd., Haidian District, BJ 100193, People's Republic of China

[5]USDA, Agricultural Research Service, Poultry Production and Product Safety Research Unit, Fayetteville, AR 72701, USA

[6]United States Department of Agriculture, Agricultural Research Service, Southeast Area, Stoneville, MS 38776, USA

Corresponding author: Xiaomao Lin (xlin@ksu.edu)

Text S1. Phenological date of maize

Phenological dates for both planting and harvest of maize for each specific location-year were collected from university maize hybrid field performance tests for 2000 through 2020. Silking and maturity dates were not available. Therefore, we used the ratio of growing degree days (GDD)[1] between planting-silking and planting-harvest calculated based on phenological data of the United States Department of Agriculture's National Agricultural Statistics Service (USDA-NASS), and daily temperature data from the Global Historical Climatological Network (GHCN)[2] to estimate silking dates by: 1) calculating state-level maize phenological dates from USDA-NASS data (Note: Because the NASS Crop Progress Report is issued only on a weekly basis in a progress percentage, we interpolated the weekly crop progress into daily data using the Piecewise Cubic Hermite Interpolating Polynomial (PCHIP) approach[3] and estimated the dates of planting, silking, and harvest defined as dates when the crop progress reached 50% completion at each phenological progress across the state (Supplementary Fig. 21); 2) estimating separately $GDD_{PT\text{-}SK,\ USDA}$ and $GDD_{PT\text{-}HV,\ USDA}$; 3) calculating GDD between planting and harvest dates based on recorded data at field sites ($GDD_{PT\text{-}HV,\ OBS}$) similar to step 2; 4) calculating GDD between planting and silking dates required for specific site-year maize fields ($GDD_{PT\text{-}SK,\ OBS}$) using the equation below; and 5) estimating silking date based on $GDD_{PT\text{-}SK,\ OBS}$ and daily temperature data. The same steps were also used to estimate maturity date. Note that we assumed the ratio of $GDD_{PT\text{-}HV,\ USDA}$ to $GDD_{PT\text{-}SK,\ USDA}$ is the same among sites within a state for a specific year.

$$GDD_{PT-SK,OBS} = \frac{GDD_{PT-HV,OBS} \times GDD_{PT-SK,USDA}}{GDD_{PT-HV,USDA}}$$

To clarify the calculated process, we estimated SK and MT by taking the Belleville site in Illinois (IL) as an example (Supplementary Fig. 4). Firstly, based on state-level phenological data in IL from USDA-NASS (Supplementary Fig. 4a), we calculated growing degree days (GDD; ^{o}C days) for three phenological periods: PT-SK; PT-MT; and PT-HV (Supplementary Fig. 4b). The GDD was calculated through an adjusted daily air temperature [$GDD = \sum_i (T_i^{adj} - 10)$]. T_i^{adj} is daily adjusted temperature (^{o}C) on day i, which was calculated by (1) imposing a maximum of 30°C and a minimum of 10°C on the daily minimum and maximum temperature, and (2) taking the average of these adjusted minimum and maximum temperatures[4]. The "10" in the equation refers to base temperature for maize to growth. Second, we calculated the state-level ratio of GDD for PT-SK and PT-MT relative to total GDD from PT to HV ($GDD_{PT\text{-}HV}$) (Supplementary Fig. 4c).

Thirdly, we used specific site-year PT and HV from field trials (Supplementary Fig. 4d) to calculate GDD_{PT-HV} for Belleville (Supplementary Fig. 4e). Fourth, assuming that the GDD ratio for a specific phenological period is consistent across sites within a state, we then estimated GDD_{PT-SK} and GDD_{PT-MT} for the Belleville site (Supplementary Fig. 4f) by multiplying the state-level GDD ratio (Supplementary Fig. 4c) with Belleville's GDD_{PT-HV} (Supplementary Fig. 4e). Finally, we estimated the silking and maturity dates based on the estimated GDD requirements (Supplementary Fig. 4g). To test the accuracy of our approach, we compared the estimated with available observed silking dates from field sites. The estimates showed a bias of 5 days (root mean square error; RMSE) (Supplementary Fig. 5), indicating the reliability of our estimation method.

Text S2. The category of maize hybrids: low-, median-, and high-yielding hybrids

We categorized maize hybrids into three groups (low-, median-, and high-yielding hybrids) for each site-year based on local percentile thresholds of maize yields. This approach ensures that hybrids of all three groups at a given site-year experience the same agricultural management practices and environments. Consequently, all three hybrids groups for each site-year appeared in each single year from 2000 to 2020. To clarify our classification approach, we used the year 2000 at the Belleville site as an example (Supplementary Fig. 22). In this case, we had yield data for 63 hybrids. We first calculated the 25th and 75th percentiles of hybrid yield for this site-year. Next, we divided hybrids into three groups: low-yielding (below the 25th percentile), median-yielding (between the 25th and 75th percentiles), and high-yielding (above the 75th percentile). We repeated this process for each site-year. Therefore, we had data from three groups from 2000 to 2020 (Supplementary Fig. 6).

Text S3. The selection of vapor pressure deficit threshold

To determine the vapor pressure deficit (VPD) thresholds selected, we employed a piecewise linear regression approach following Schlenker and Roberts (2009)[5]. This approach allows for one threshold per phenological stage, thus detecting potential nonlinear yield response to VPD. We estimated a range of candidate thresholds and the performance of the corresponding models. The final thresholds were selected based on the lowest Akaike Information Criterion (AIC) value. AIC is a widely accepted metric for model comparison, where lower values indicating a better fit[6].

When the difference in AIC values between two models is less than 2, it suggests that the models perform similarly well[7].

The candidate threshold window was determined using the distribution of daily VPD during each phenological stage. We restricted the threshold range from the 50th to 90th percentile range (Supplementary Fig. 7a,b) to ensure coverage from median to extreme stress levels while minimizing noise from the tails of the distribution. This corresponds to a range of 1.3-2.1 kPa for the vegetative (VEG) stage and 1.3-1.9 kPa for the grain filling stage (GFP). To improve computation efficiency, we discretized this range into 49 threshold pairs, ranging from 1.3 kPa to 1.9 kPa in increments of 0.1 kPa (Supplementary Fig. 7c). For each pair, we constructed two measures of VPD exposures (below and above threshold) for each phenological stage and re-estimated performance of the full model. The optimal VPD thresholds (the lowest AIC) were estimated to be 1.4 kPa for VEG and 1.3 kPa for GFP (Supplementary Fig. 7d). This approach also worked well in previous studies to estimate temperature threshold impacting U.S. wheat yield[8].

Text S4. Procedures to estimate breeding-driven yield gains under varying stress conditions. To estimate breeding-driven yield gains under varying stress conditions, we used low-yielding hybrids (LYH) as an example (Supplementary Fig. 23). We first calculated the average yield of hybrids for each site-year (Supplementary Fig. 23a), representing site-year environment index. For each year, we categorized all sites into five environmental stress levels based on yield percentiles (10th, 25th, 75th, and 90th percentiles) (Supplementary Fig. 23b-c), and calculated the average yield across sites for each environmental stress level (Supplementary Fig. 23d). Next, we fitted a linear regression of yield ***vs.*** years for each stress level (Supplementary Fig. 23d) and extracted the slope and its 95% CI (Supplementary Fig. 23e). Lastly, we plotted the estimated trends and their CIs for all environmental stress levels (Supplementary Fig. 23f).

Text S5. Change in drought resistance over breeding for different production conditions

To determine how breeding advancements may have influenced drought resistance of maize yield under different production conditions, we conducted a linear regression between trial year and average yield for each state (Supplementary Fig. 18a). Based on fitted yield trend and its 95% confidence interval (CI), we then categorized years into three groups: good, normal, and bad years. Specifically, years where observed mean yield exceeded the upper CI were classified as good years

(blue points; Supplementary Fig. 18a), those below the lower CI as bad years (red points; Supplementary Fig. 18a), and those within the CI range as normal years (gray points; Supplementary Fig. 18a). Next, we split the original dataset into three production conditions (Supplementary Fig. 18b) and re-estimated the VPD sensitivity for each data subset using the same linear mixed-effects modeling framework as described in the Main text (Supplementary Fig. 18c).

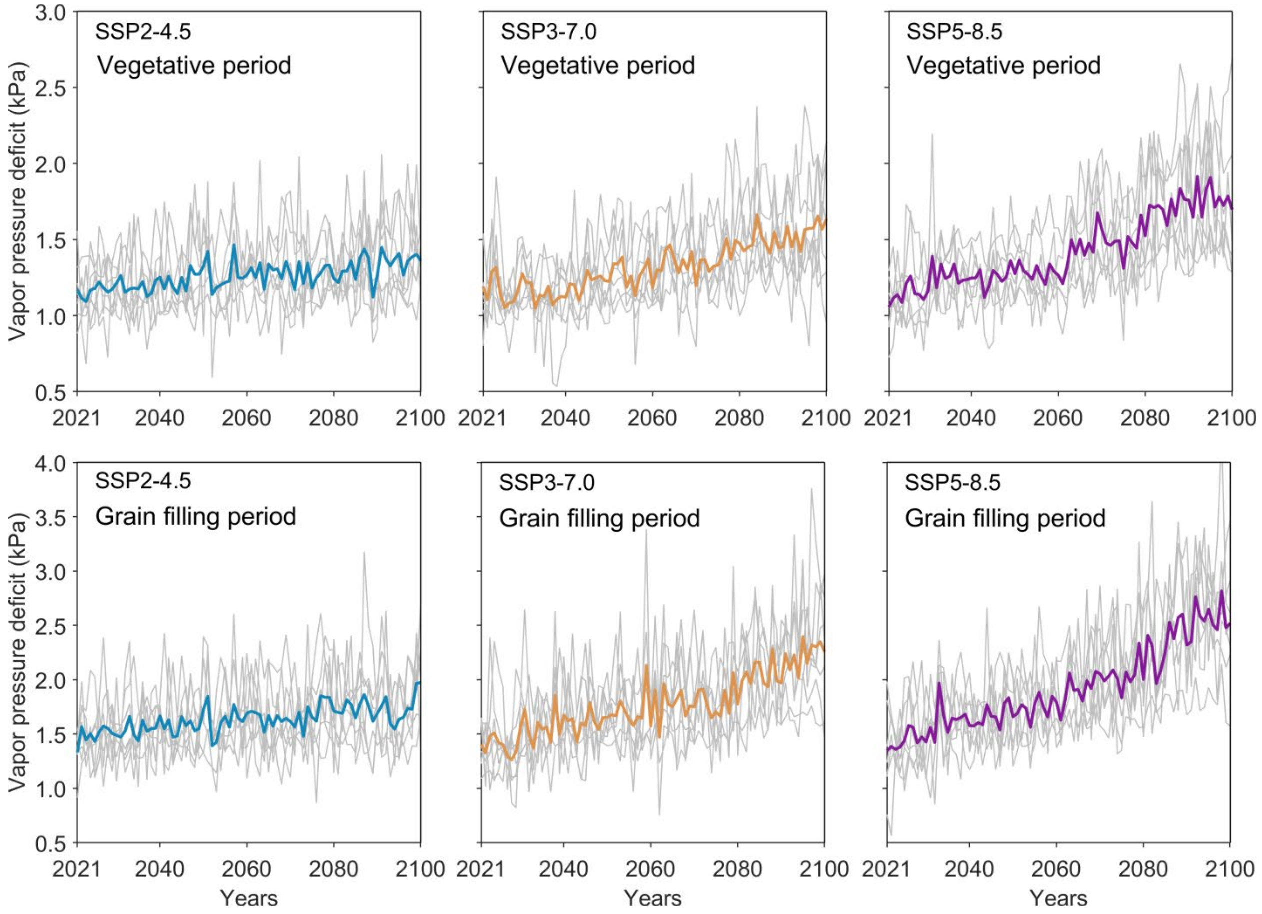

Fig. S1 | Time series of the projected vapor pressure deficit during the maize vegetative (from sowing to silking) and grain filling period (from silking to maturity) under three shared socioeconomic pathways (SSP). Solid color lines refer to the ensemble average from seven climate models (gray lines).

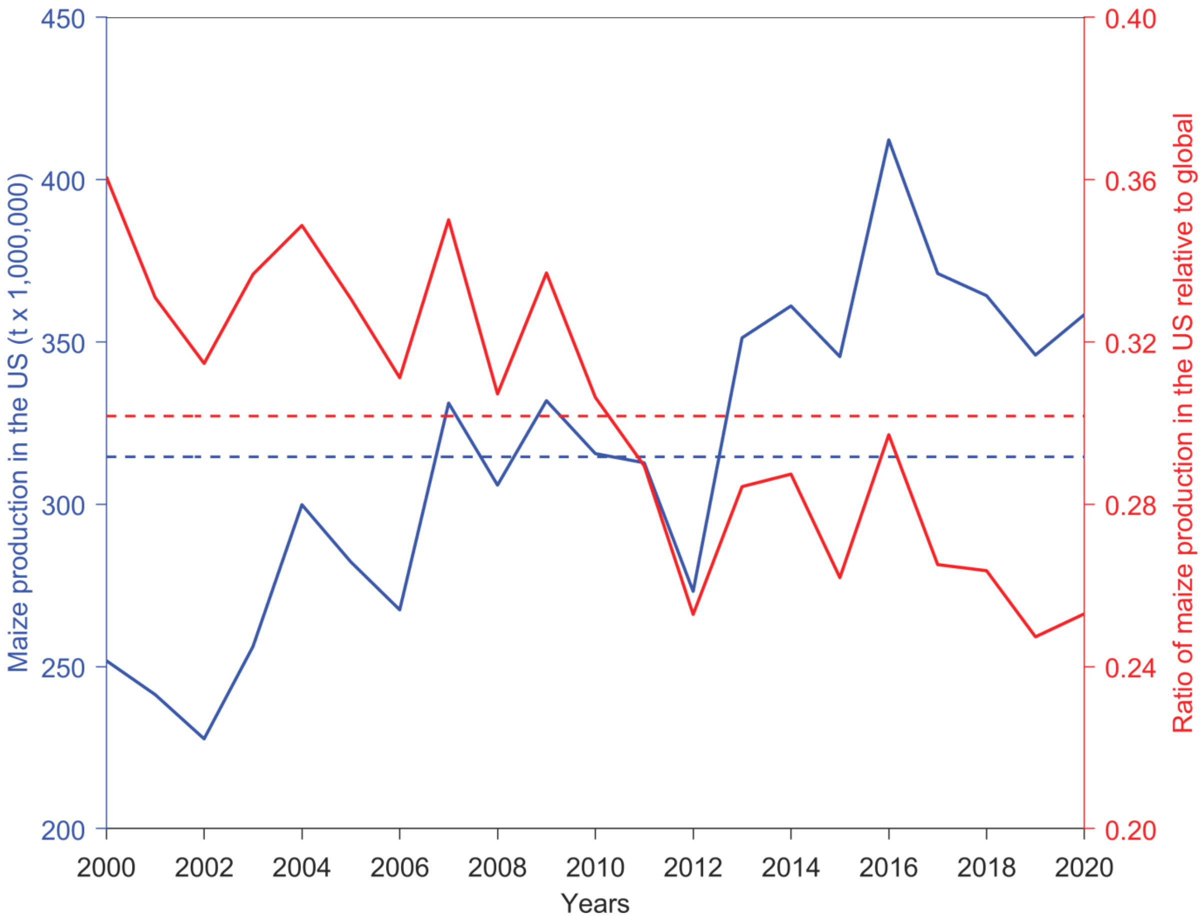

Fig. S2 | Time series of maize production in the U.S. and the corresponding annual ratio of production relative to global totals from 2000 to 2020. The blue line refers to maize production in the U.S. (metric tons) and the red line refers to the ratio of production in the U.S. relative to global production. Horizonal lines represent average values. Data source is (https://www.fao.org/faostat/en/#data/QCL).

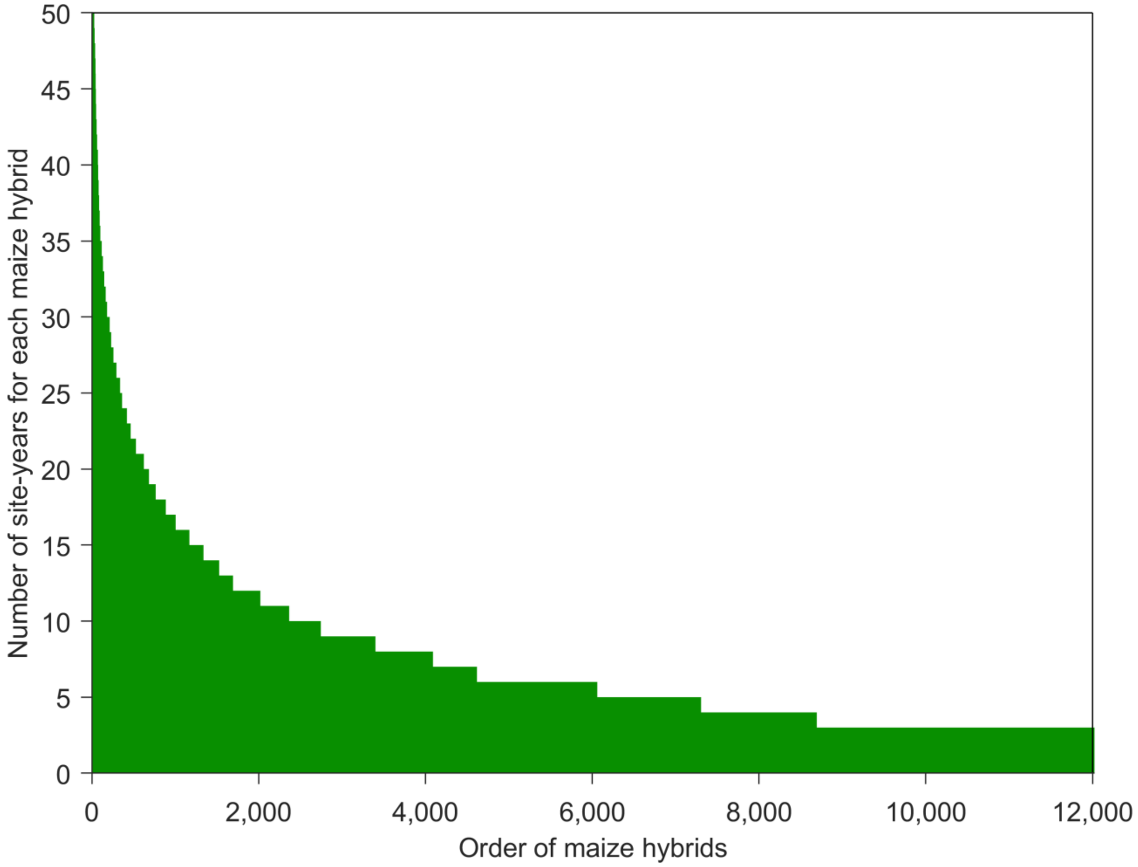

Fig. S3 | Hybrids ranked by the total number of site-years in which they appear. The height of each bar is the total number of site-years in which each hybrid appeared.

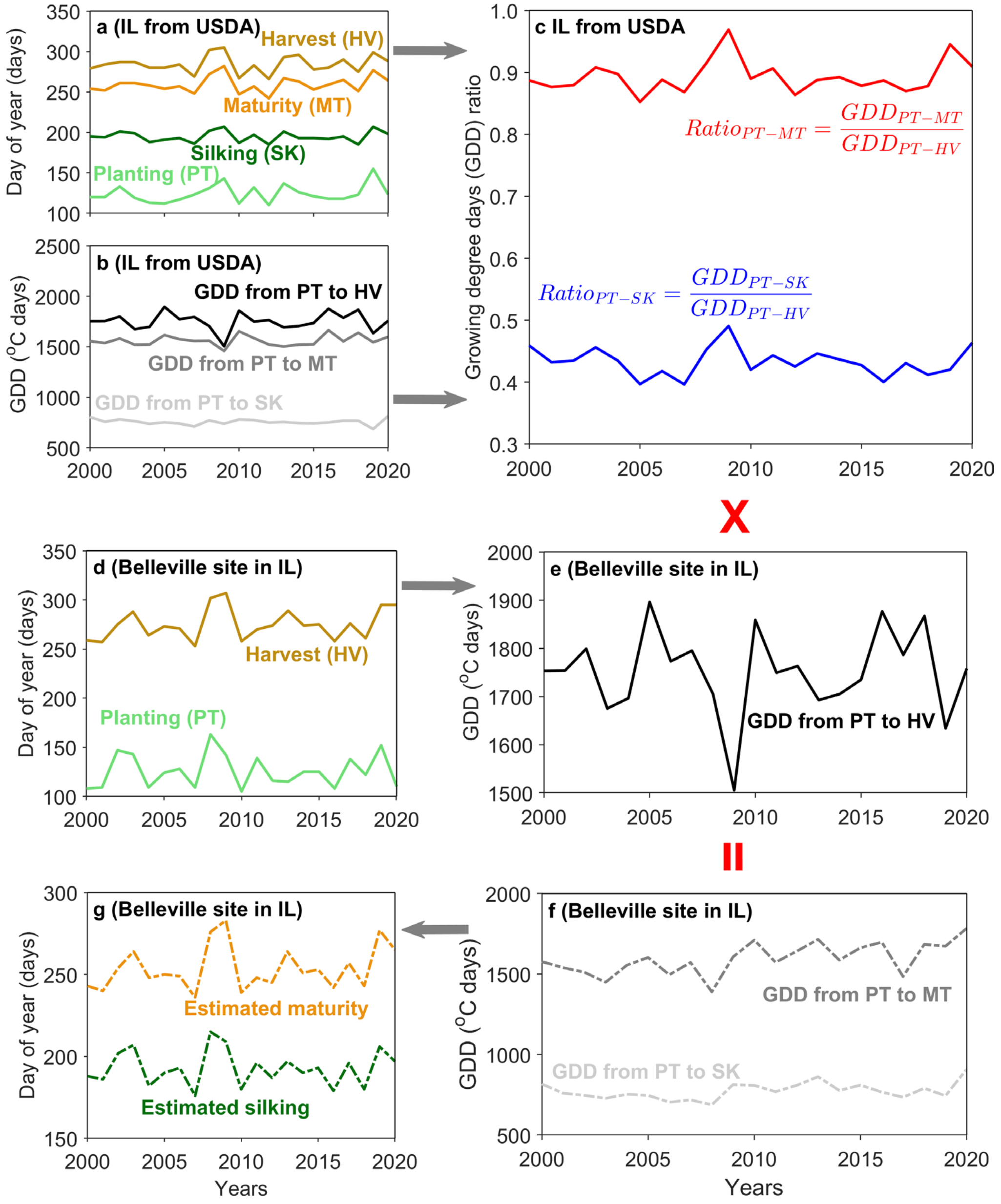

Fig. S4 | Flow of estimating silking and maturity dates from 2000 to 2020 using the Belleville site in Illinois (IL) as an example. a, Observed state-level phenological periods, derived from the USDA dataset. **b**, State-level growing degree days (GDD) required for specific phenological periods, including from planting (PT) to silking (SK) ($GDD_{PT\text{-}SK}$), from PT to maturity (MT) ($GDD_{PT\text{-}MT}$), and from PT to harvest (HV) ($GDD_{PT\text{-}HV}$). **c**, The ratio of GDD for specific phenological periods relative to $GDD_{PT\text{-}HV}$. **d**, Observed PT and HV. **e**, GDD required from PT and HV. **f**, Estimated $GDD_{PT\text{-}SK}$ and $GDD_{PT\text{-}MT}$ based on panel **c** and **e**. **g**, Estimated annual silking and maturity dates.

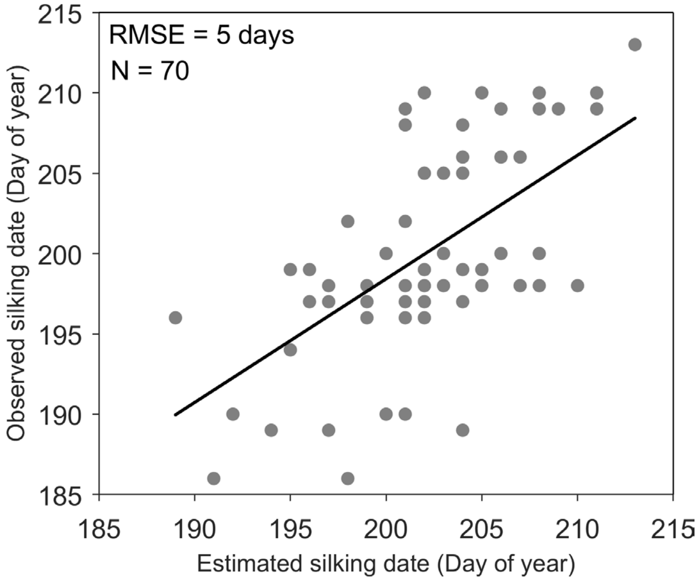

Fig. S5 | Estimated *vs.* observed silking date of maize from field sites in Ohio from 2000 to 2012. The solid line refers to the fitted line. RMSE refers to root mean square error, showing the estimated performance. N represents the number of samples.

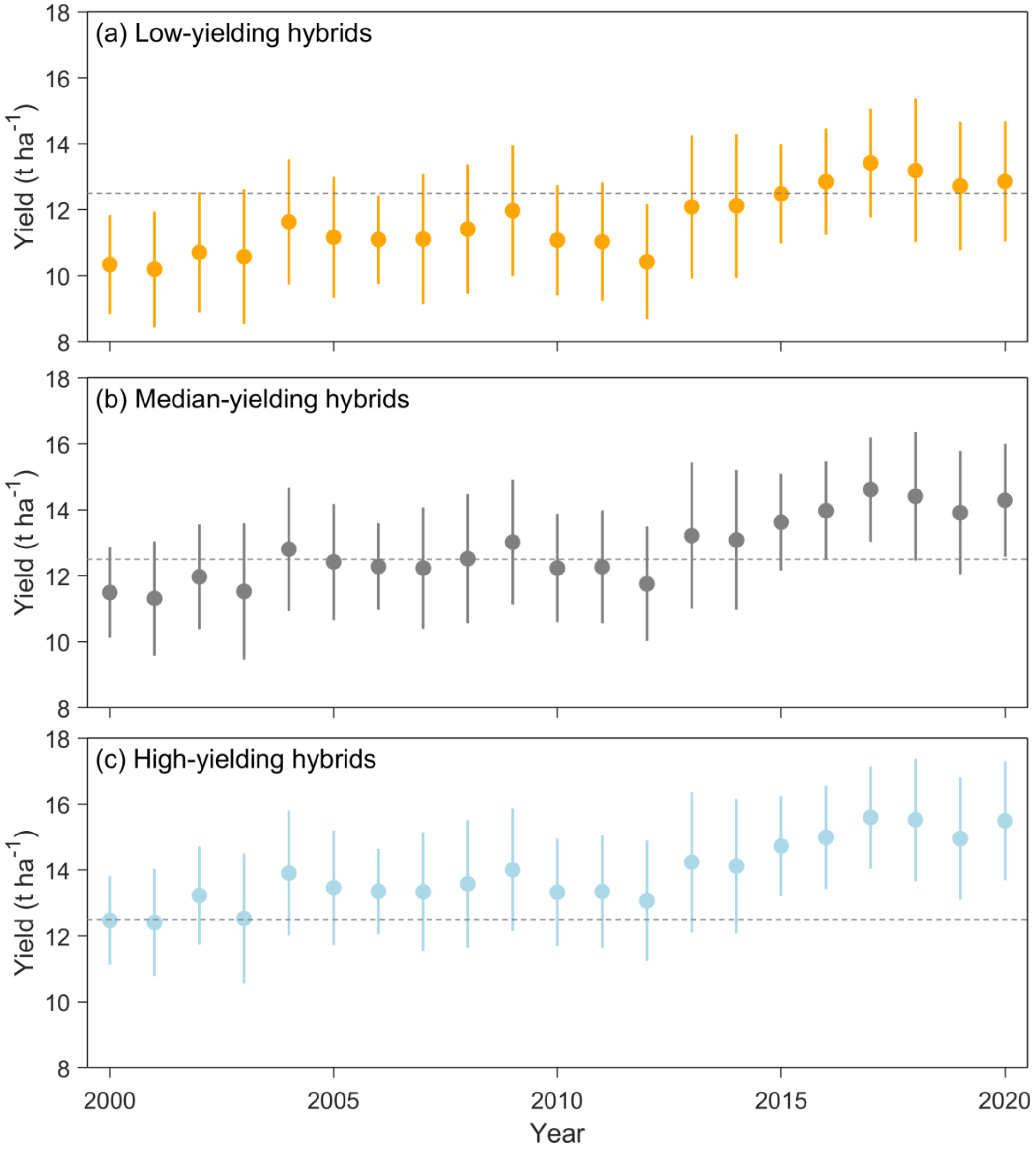

Fig. S6 | Time series of maize yield from 2000 to 2020 for hybrids with low-yielding (a), median-yielding (b), and high-yielding (c) hybrids. The dots are average yield, and the vertical lines represent one standard deviation. The grey dashed line in each panel is the average across all types for 2000 through 2020. The annual mean sample sizes for three yielding types are 1,071, 2,246, and 1,068, respectively.

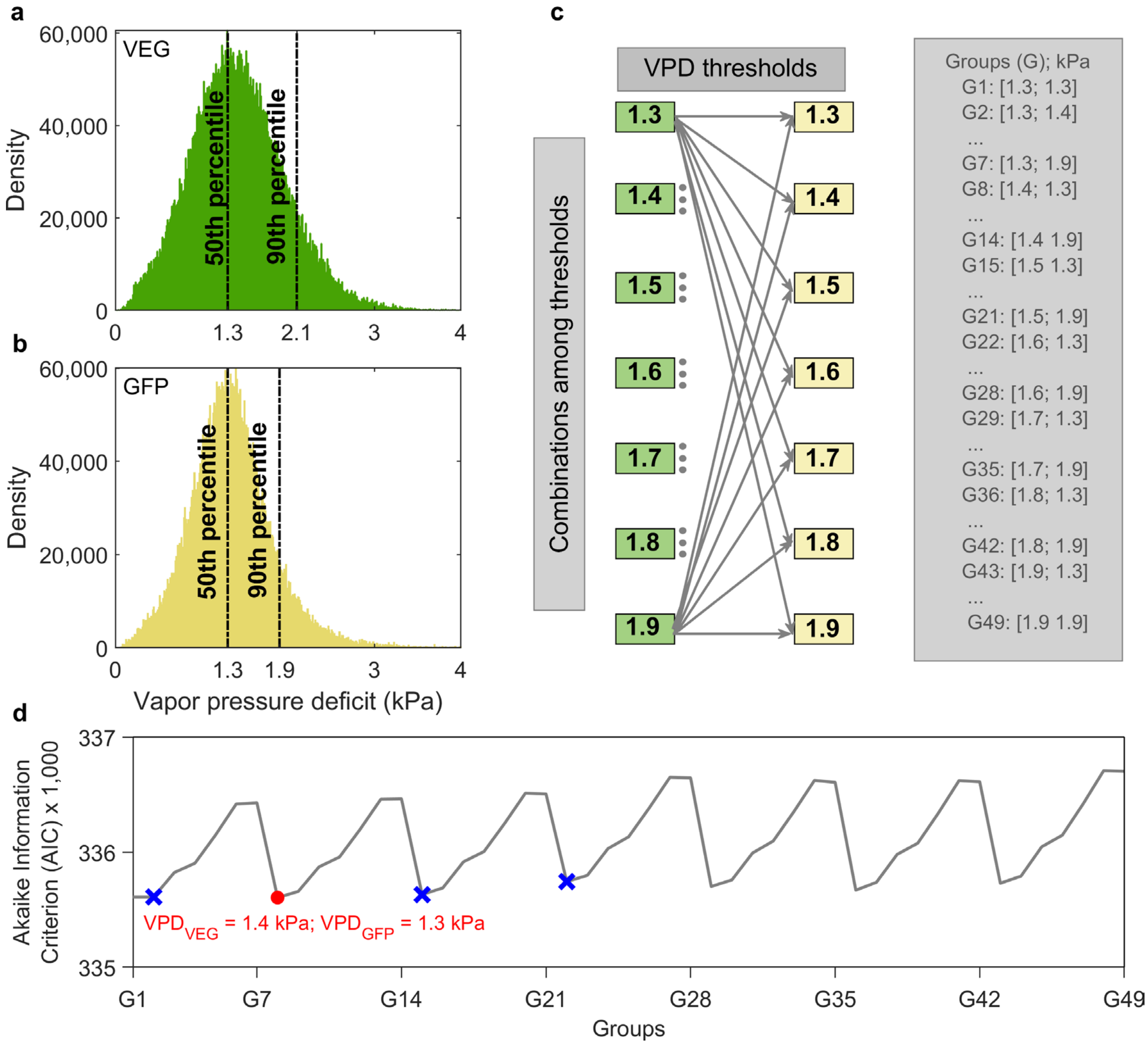

Fig. S7 | Flow chart for the procedure of selecting the vapor pressure deficit (VPD) threshold. **a**, The distribution of daily VPD during the vegetative stage (VEG). Vertical dashed lines represent the selection window of the VPD threshold spanning from the 50th to the 90th percentile. **b**, Same as panel **a**, but for the grain filling period (GFP). **c**, Matrix of 49 paired VPD threshold combinations for VEG; GFP. **d**, The Akaike Information Criterion (AIC) values for each threshold group G1 to G49.

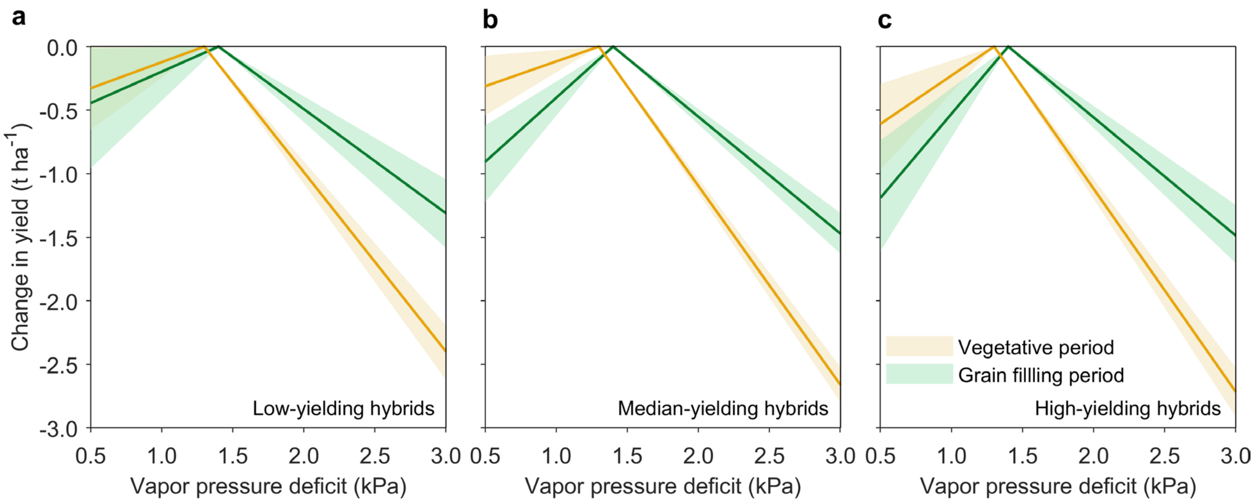

Fig. S8 | Nonlinear effect of the vapor pressure deficit (VPD) on maize yield during the vegetative (from sowing to silking) and grain filling periods (from silking to maturity) for low-yielding (a), median-yielding (b), and high-yielding (c) types. Slopes to the left and right of the optimal VPD threshold indicate the sensitivity of VPD below and above the threshold. The solid lines correspond to the ensemble average with colored shadow areas displaying the 2.5th – 97.5th percentile range of 1,000 bootstraps.

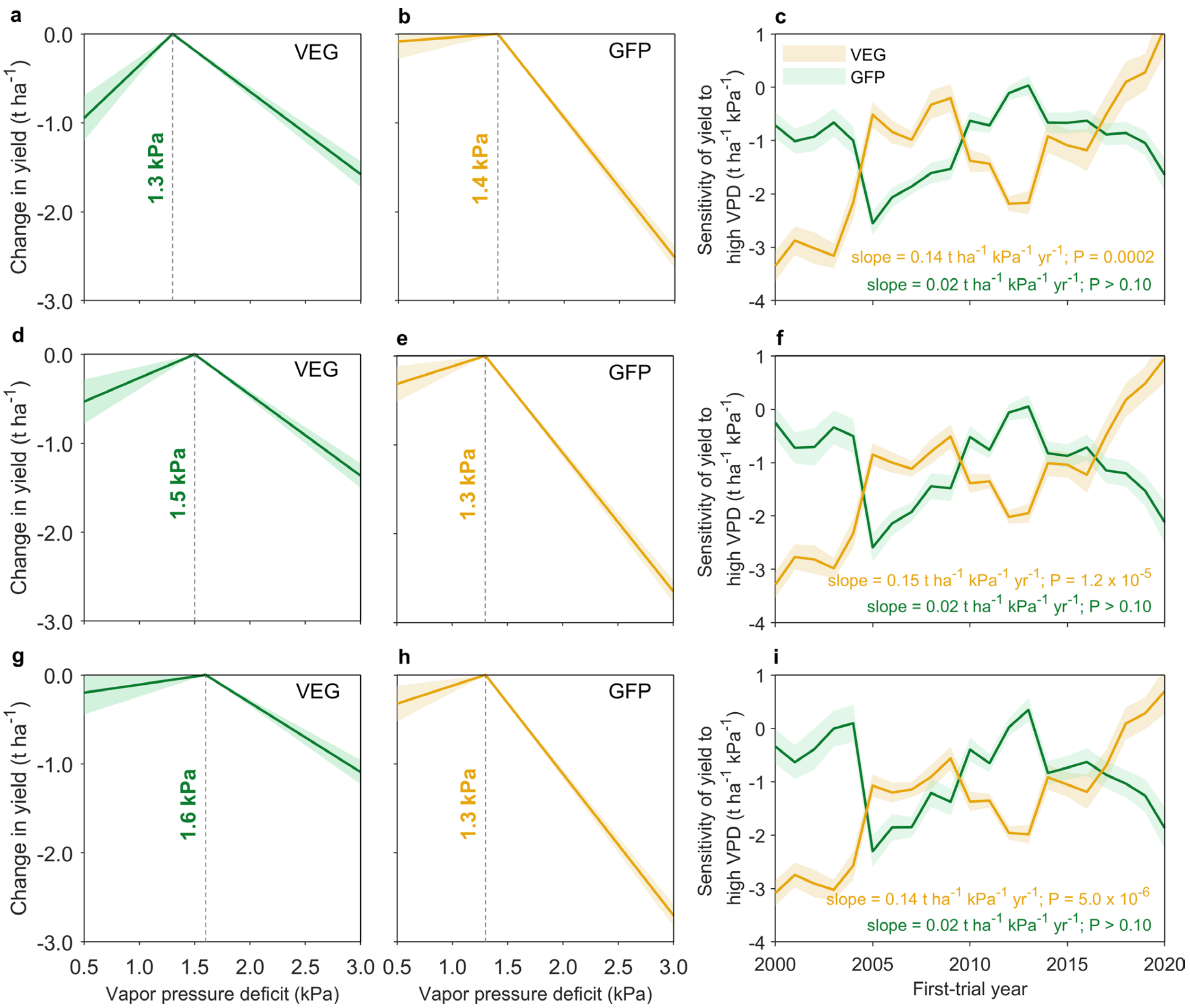

Fig. S9 | The nonlinear effect of the vapor pressure deficit (VPD) on maize yield and the estimated change in drought resilience of hybrid advancement during the vegetative (VEG) (green) and grain filling period (GFP) (orange) using three additional combinations of VPD thresholds, including [1.3 kPa (VEG), 1.4 kPa (GFP)] (a-c); [1.5 kPa (VEG), 1.3 kPa (GFP)] (d-f); and [1.6 kPa (VEG), 1.3 kPa (GFP)] (g-i). Slopes to the left and right of the VPD threshold (vertical dashed line) in panels a,b,d,e,g,h indicate sensitivity to VPD below and above the threshold. The solid lines correspond to the ensemble average with colored shadow areas displaying 2.5th – 97.5th percentile range of 1,000 bootstraps. The solid lines (panels c,f,i) represent the ensemble average with colored shadow areas displaying 2.5th – 97.5th percentile range of 1,000 bootstraps. Trends were estimated using linear regression (two-sided test). No adjustment for multiple comparisons was applied.

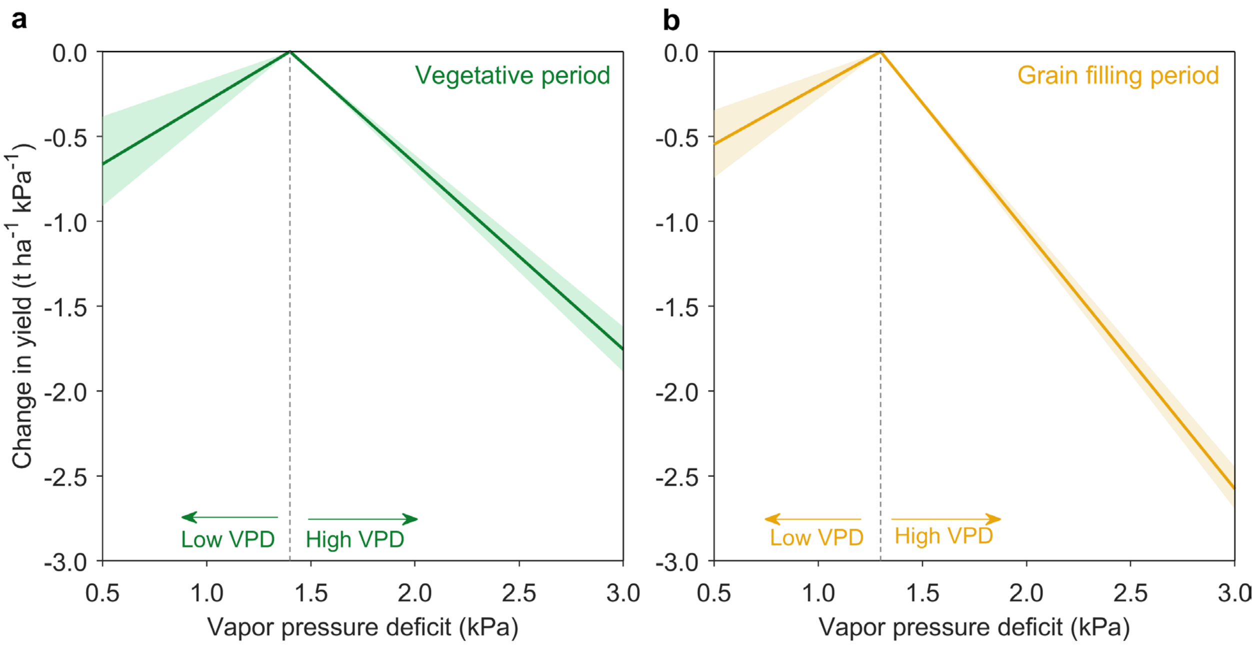

Fig. S10 | The nonlinear effect of the vapor pressure deficit (VPD) on maize yield during growing season based on data from the western and central U.S. corn belt. a, Effect of VPD on yield during the vegetative period (from planting to silking). Slopes to the left and right of VPD threshold (vertical dashed line) indicate the sensitivities to VPD below and above the threshold. The solid lines correspond to the ensemble average with colored shadow areas displaying the 2.5^{th} – 97.5^{th} percentile range of 1,000 bootstraps. **b**, Same as **a** but for the grain filling period (from silking to maturity).

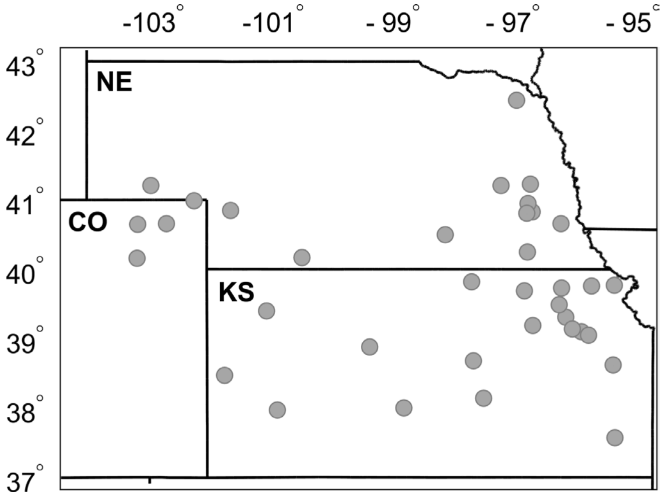

Fig. S11 | Map of the study region across the western U.S. corn belt with circles representing field sites (Kansas, KS; Colorado, CO; and Nebraska, NE). Data are derived from rainfed, university field performance tests of maize hybrids from 2000 to 2020.

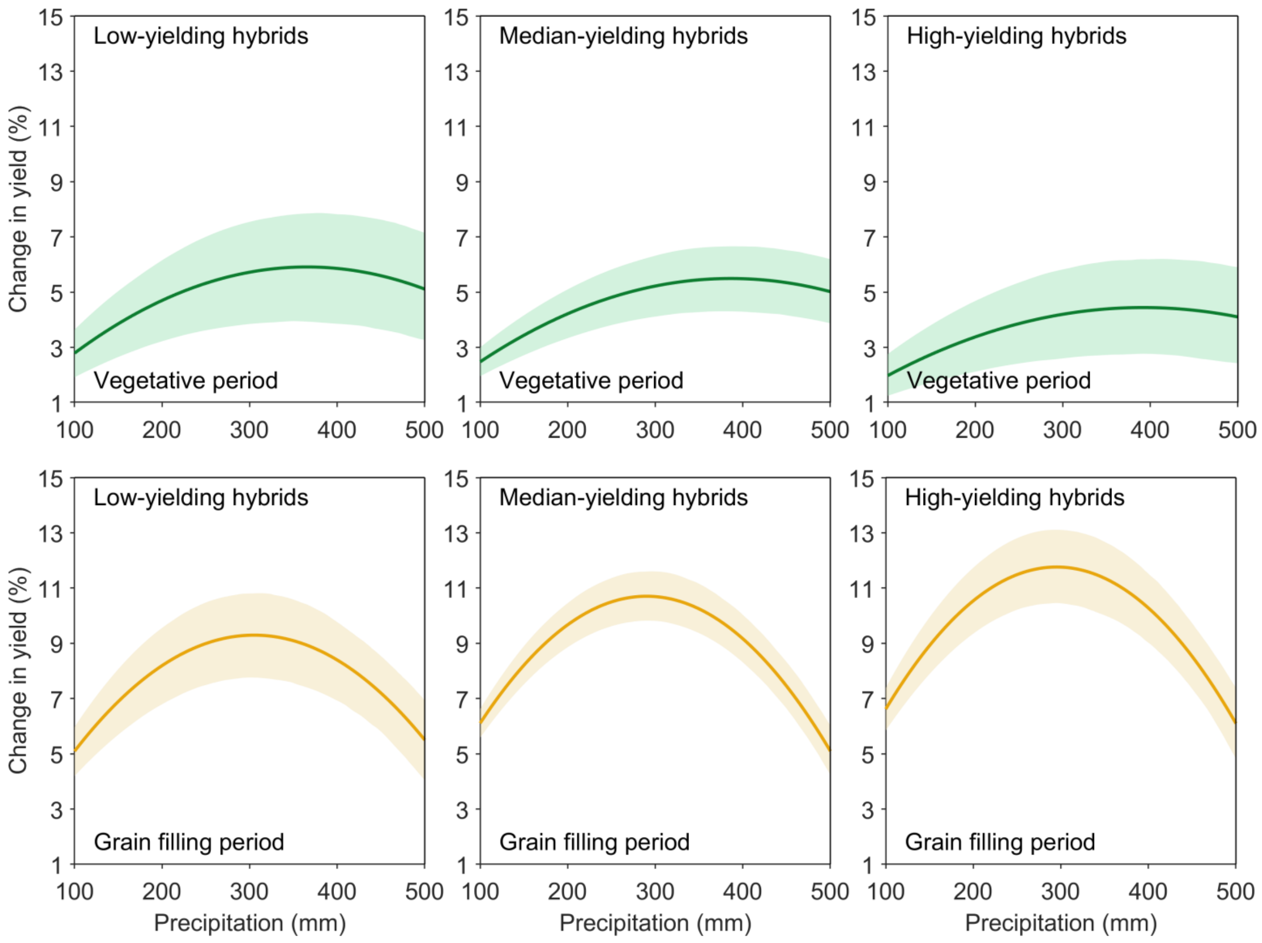

Fig. S12 | Nonlinear impact of precipitation on maize yield during the vegetative (VEG, sowing to silking) and grain filling period (GFP, silking to maturity) for three yielding types. Solid lines refer to average effects. The shadowed areas correspond to the 2.5th – 97.5th percentile range of 1,000 bootstraps.

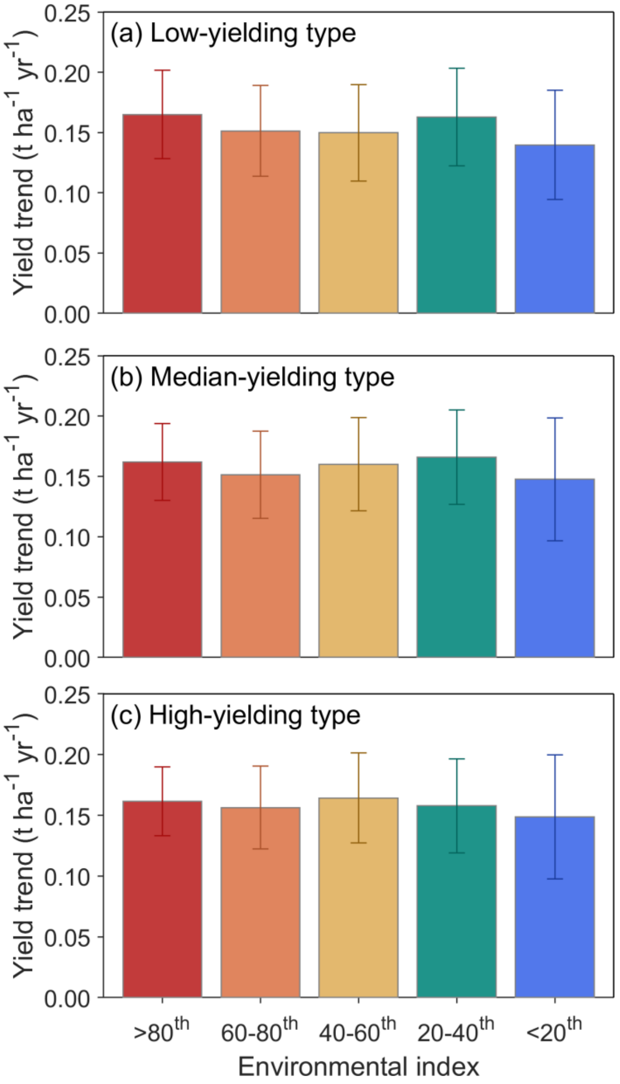

Fig. S13 | Trend of maize yield across five environmental index levels from 2000 to 2020 for low-yielding (a), median-yielding (b), and high-yielding (c) types. Bars refer to the mean trend, while error bars indicate the 95% confidence interval (sample size for each environmental condition = 21 years). Trends were estimated using linear regression (two-sided test). No adjustment for multiple comparisons was applied.

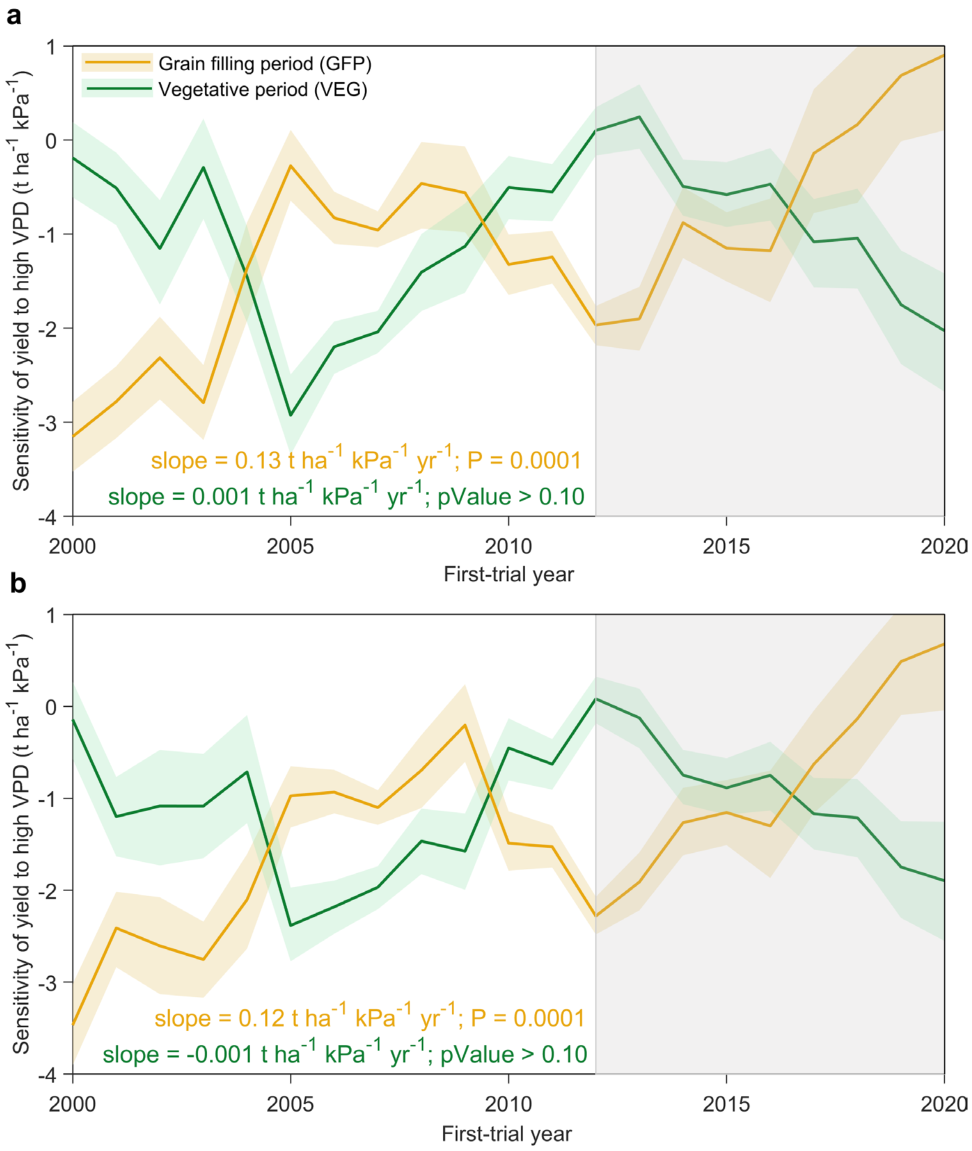

Fig. S14 | Change in sensitivity of yield to vapor pressure deficit (VPD) among different yielding types during the growing season. a, Time series in sensitivity of yield to VPD for low-yielding type during vegetative (from sowing to silking) and grain filling periods (from silking to maturity). The solid lines represent the ensemble average with colored shadow areas displaying the 2.5th – 97.5th percentile range of 1,000 bootstraps. Trends were estimated using linear regression (two-sided test). No adjustment for multiple comparisons was applied. **b,** Same as **a** but for high-yielding types.

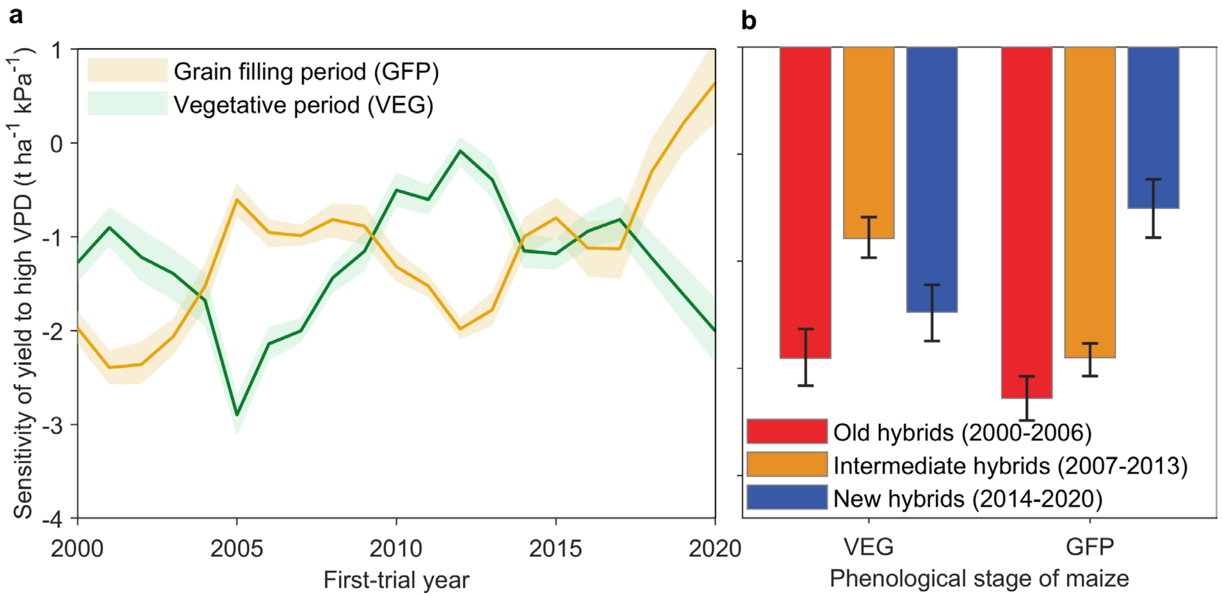

Fig. S15 | Change in sensitivity of maize yield to the vapor pressure deficit (VPD) during growing season based on data from the western and central U.S. corn belt. a, Time series in sensitivity of yield to VPD over breeding during the vegetative period (VEG; from sowing to silking) and grain filling period (GFP; from silking to maturity). The solid lines represent the ensemble average with colored shadow areas displaying the 2.5th – 97.5th percentile range of 1,000 bootstraps. **b**, The sensitivity of yield to VPD for old hybrids (2000-2006), intermediate hybrids (2007-2013), and new hybrids (2014-2020). Bars represent the ensemble average with error bars showing the 2.5th – 97.5th percentile range. The modeling sample size is 101,088.

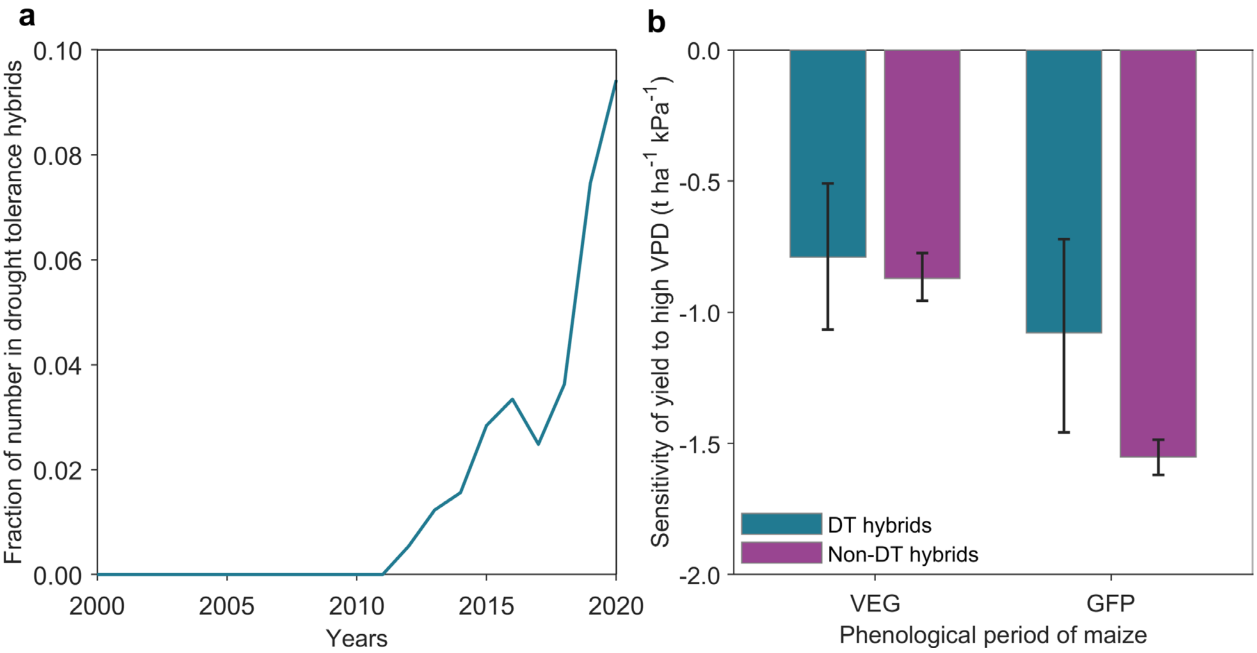

Fig. S16 | Change in number of drought-tolerant (DT) hybrids and corresponding effect on maize yield. a, Fraction in number of hybrids with DT hybrids to the number of all hybrids in the specific first-trial year. **b,** The sensitivity of yield to VPD above thresholds for DT hybrids ***vs.*** non-DT hybrids during vegetative (VEG; from sowing to silking) and grain filling periods (GFP; from silking to maturity). Bars represent the ensemble average with error bars showing the 2.5th – 97.5th percentile range. The modeling sample size is 92,096.

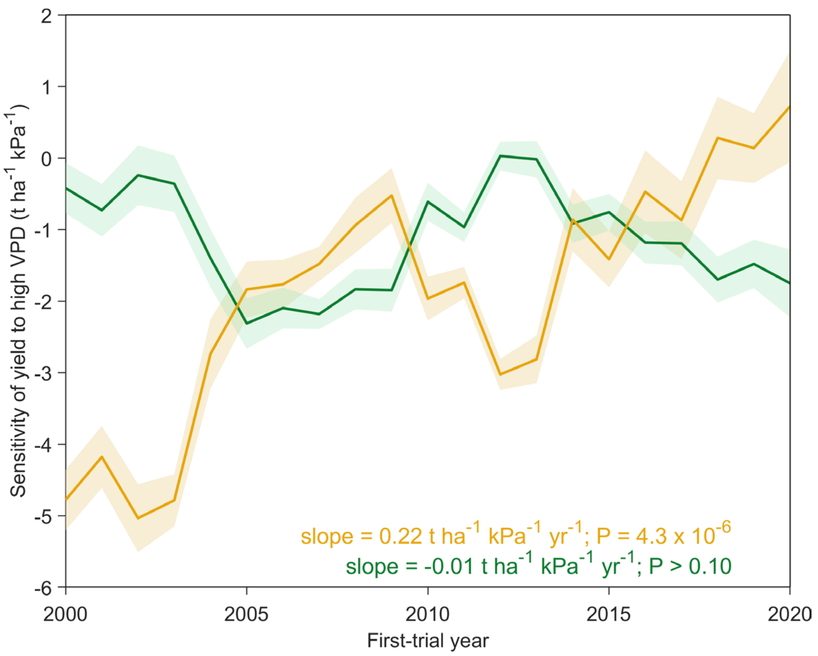

Fig. S17 | Change in sensitivity of yield to vapor pressure deficit (VPD) during vegetative (green) and grain filling periods (yellow) based on daily mean VPD[9]. The solid lines represent the ensemble average with colored shadow areas displaying the 2.5th – 97.5th percentile range of 1,000 bootstraps. Trends were estimated using linear regression (two-sided test). No adjustment for multiple comparisons was applied.

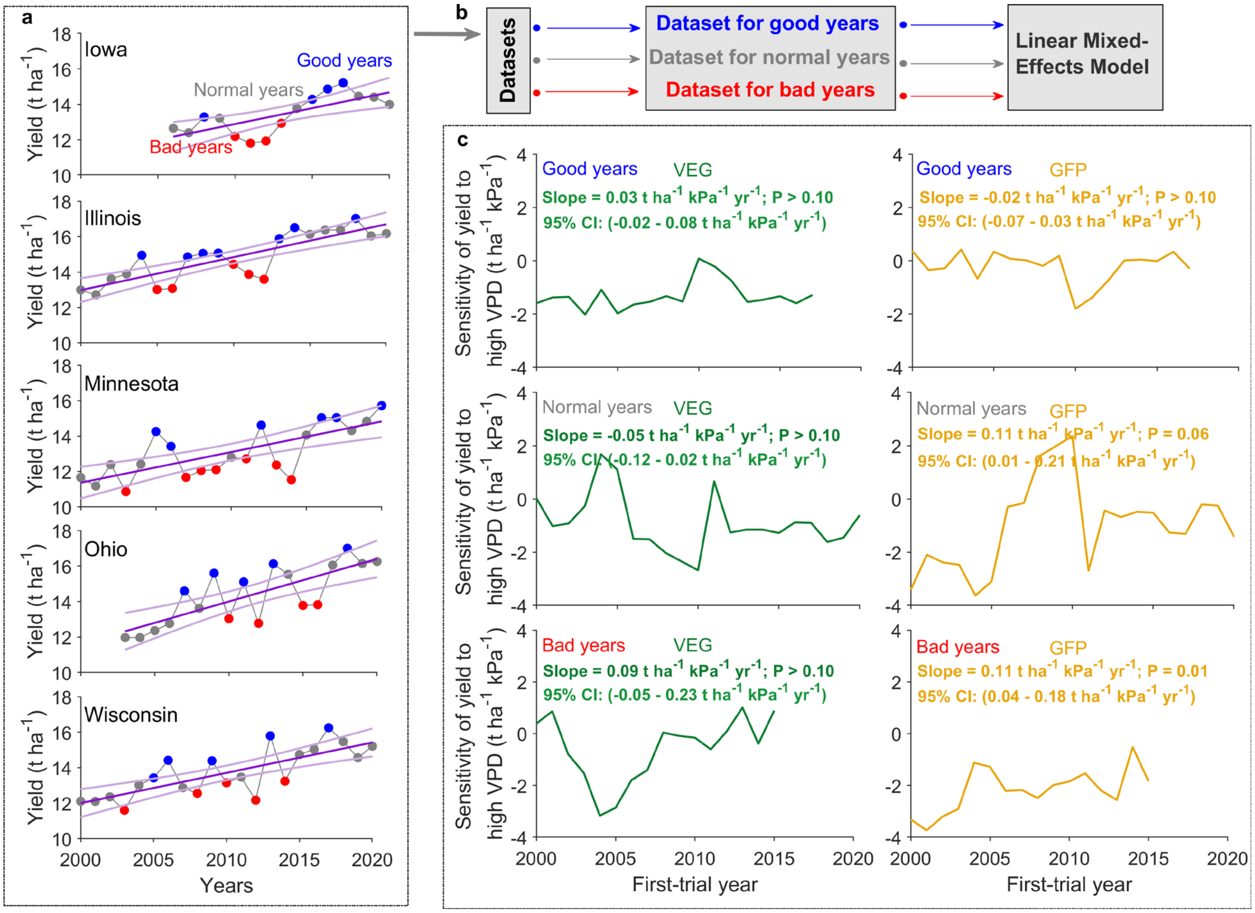

Fig. S18 | The framework to estimate the change in high vapor pressure deficit (VPD) resistance of maize yield over breeding progress under different production conditions. a, State-level time series of maize yields. Solid lines refer to regressed yields (dark purple) with a 95% confidence interval (95% CI; light purple). Blue points refer to good years, gray points are normal years, and red points represent bad years. **b**, Flowchart for data classification and modeling. **c**, Time series in sensitivity of yield to VPD during the vegetative period (VEG; from sowing to silking) and grain filling period (GFP, from silking to maturity). Trends were estimated using linear regression (two-sided test). No adjustment for multiple comparisons was applied. Regression model fit with residual degrees of freedom are 16, 18, and 14 for good, normal, and bad years, respectively.

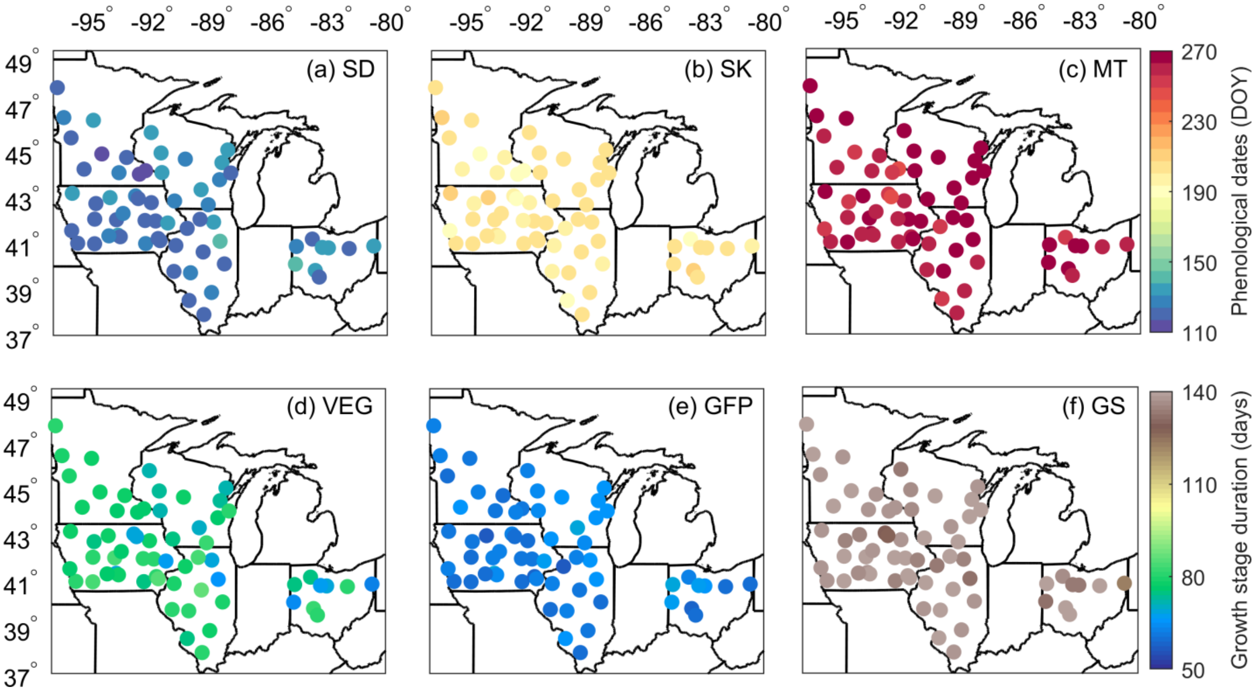

Fig. S19 | Maize phenological date and growth stage duration. a-c phenological date in day of year (DOY): sowing date (SD), silking date (SK), and maturity (MT). **d-f** growth stage duration in days: sowing to silking (VEG), silking to maturity (GFP), and sowing to maturity (GS).

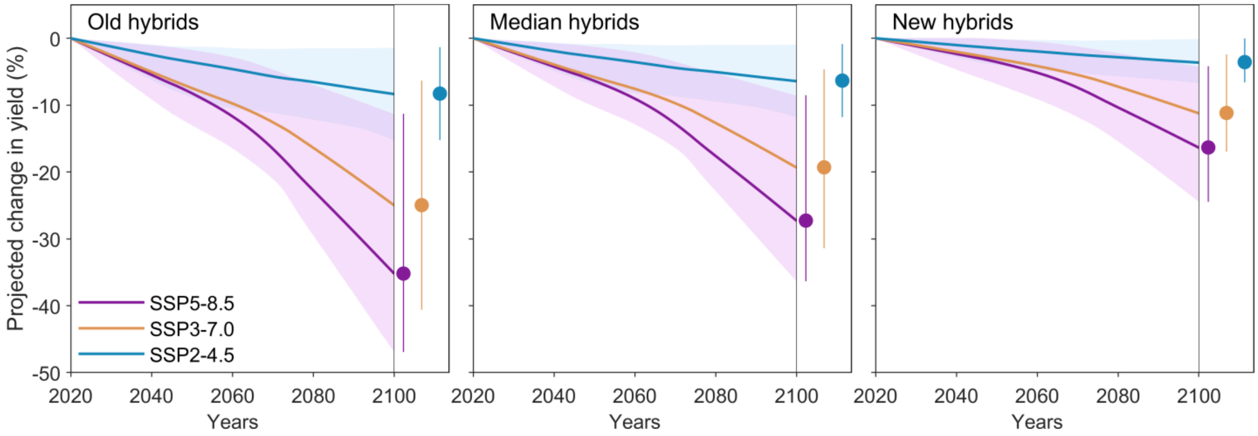

Fig. S20 | Projected yield change driven by future vapor pressure deficits over time relative to historical average for old (released from 2000 to 2006), median (released from 2007 to 2013), and new (released from 2016 to 2020) hybrids of a median-yielding type under three climate scenarios (SSPs). The solid lines highlight average projections with the colored shadow areas displaying the 2.5th – 97.5th percentile range of 1,000 bootstraps × 7 climate models (sample size = 7,000). The dots are average, and the error bars show the 2.5th – 97.5th percentile range of projected yield impacts by 2100.

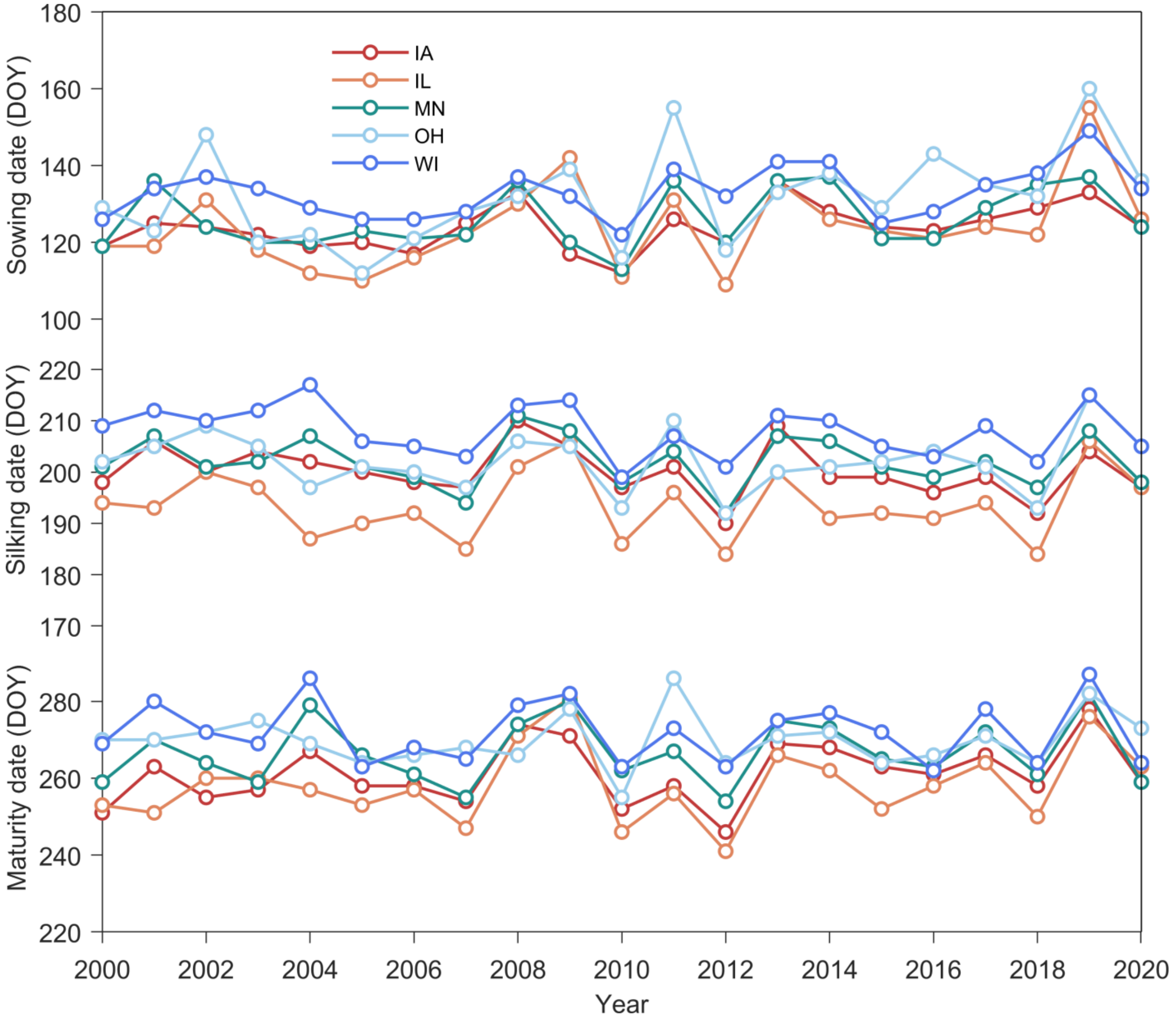

Fig. S21 | State-level time series of maize phenological data derived from the United States Department of Agriculture's National Agricultural Statistics Service including sowing, silking, and maturity in Iowa (IA), Illinois (IL), Minnesota (MN), Ohio (OH), and Wisconsin (WI).

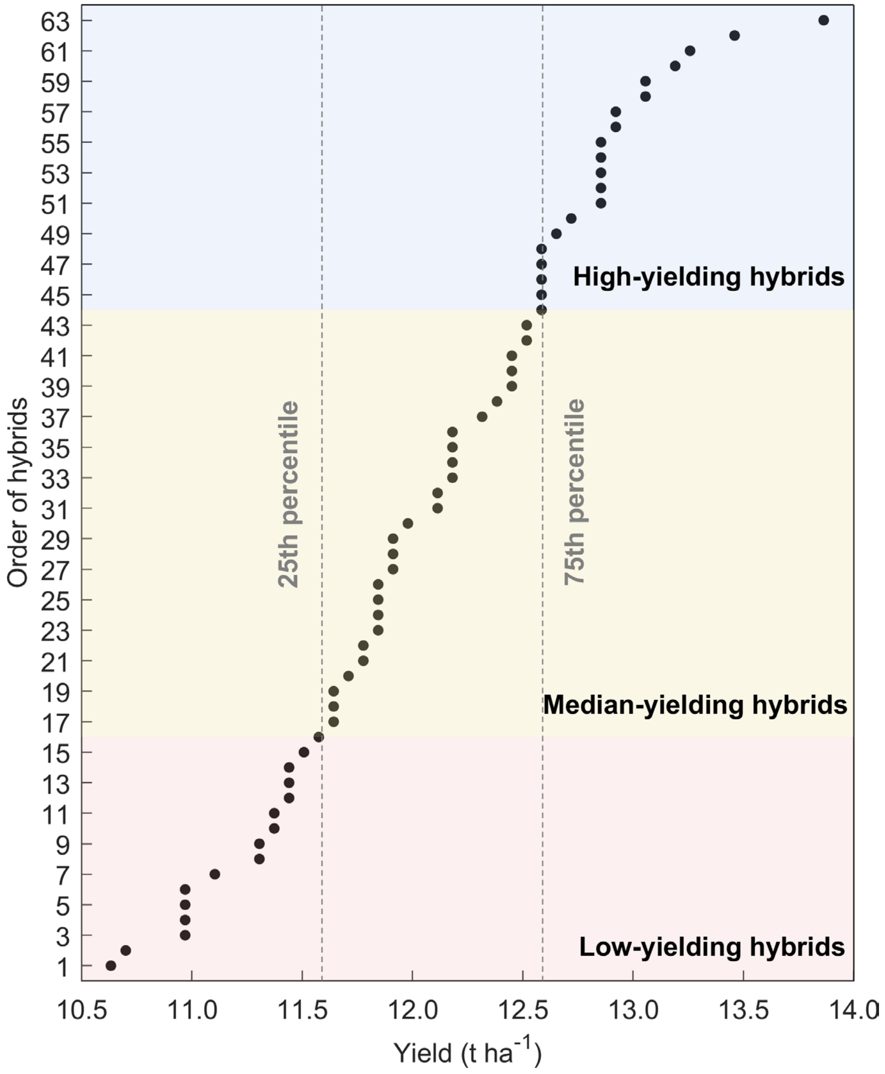

Fig. S22 | A case (year 2000 for the Belleville site) for dividing maize hybrids into three yielding types, including low-yielding, median-yielding, and high-yielding hybrids. The yield performance of 63 maize hybrids was measured in 2000 at the Belleville site. The x-axis refers to yield, and the y-axis represents hybrids. Black points are actual maize yield for each hybrid. Vertical dash lines represent the 25th and 75th percentiles for defining the boundary of three yielding classes.

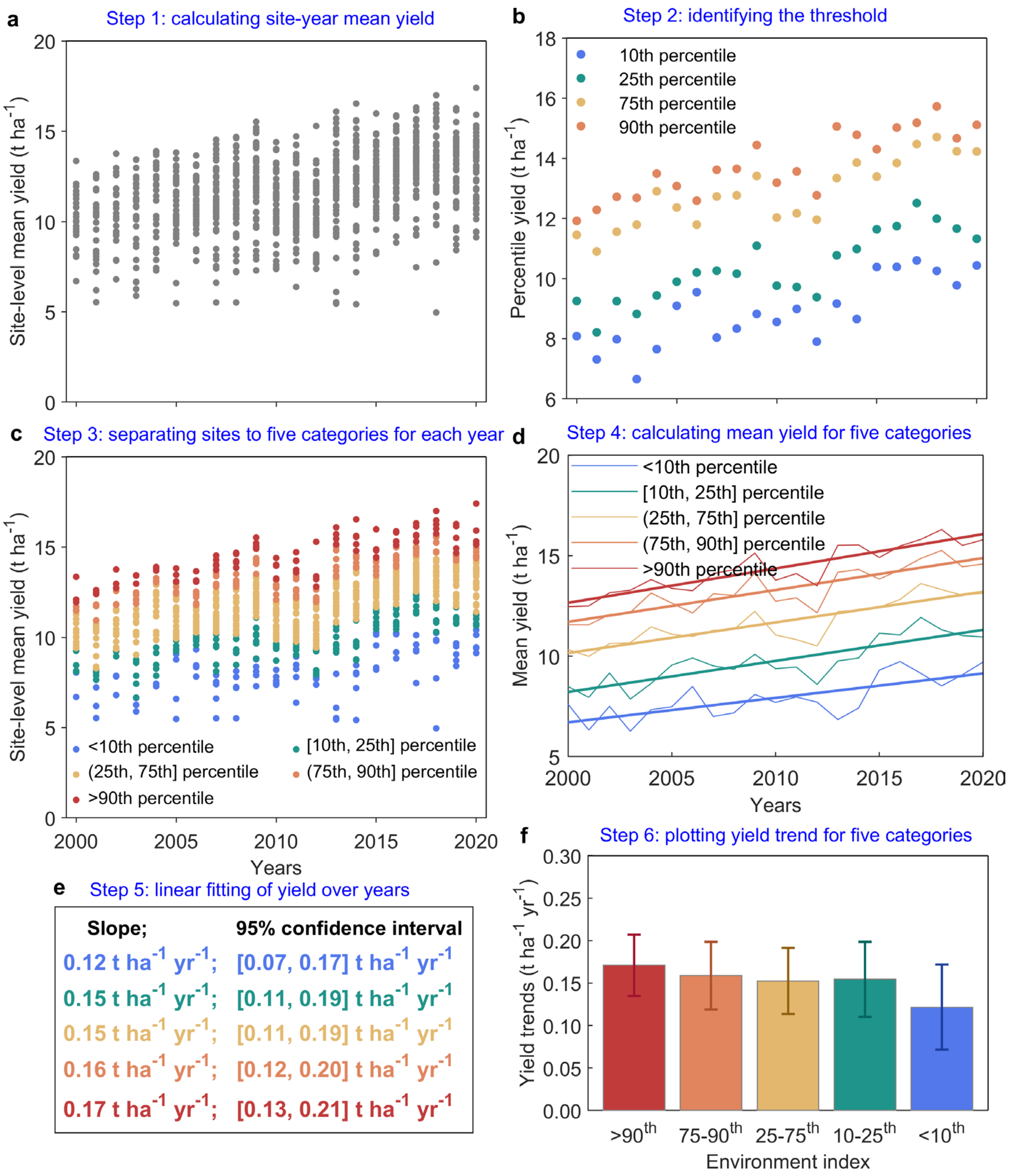

Fig. S23 | Yield trends across varying environmental stress levels. a, Site-level mean yield for each year. Points represent sites. **b**, Yield percentiles across all sites for each year. The lower percentile represents the worse environmental level. **c**, Same as panel **a**, but points were classified into specific environmental levels. **d**, Time series of mean yield over years. **e**, Estimated yield trends and their 95% confidence intervals (CIs). **f**, Yield trends and their 95% CI for all environmental stress levels (sample size for each environmental condition = 21 years). Trends were estimated using linear regression (two-sided test). No adjustment for multiple comparisons was applied.

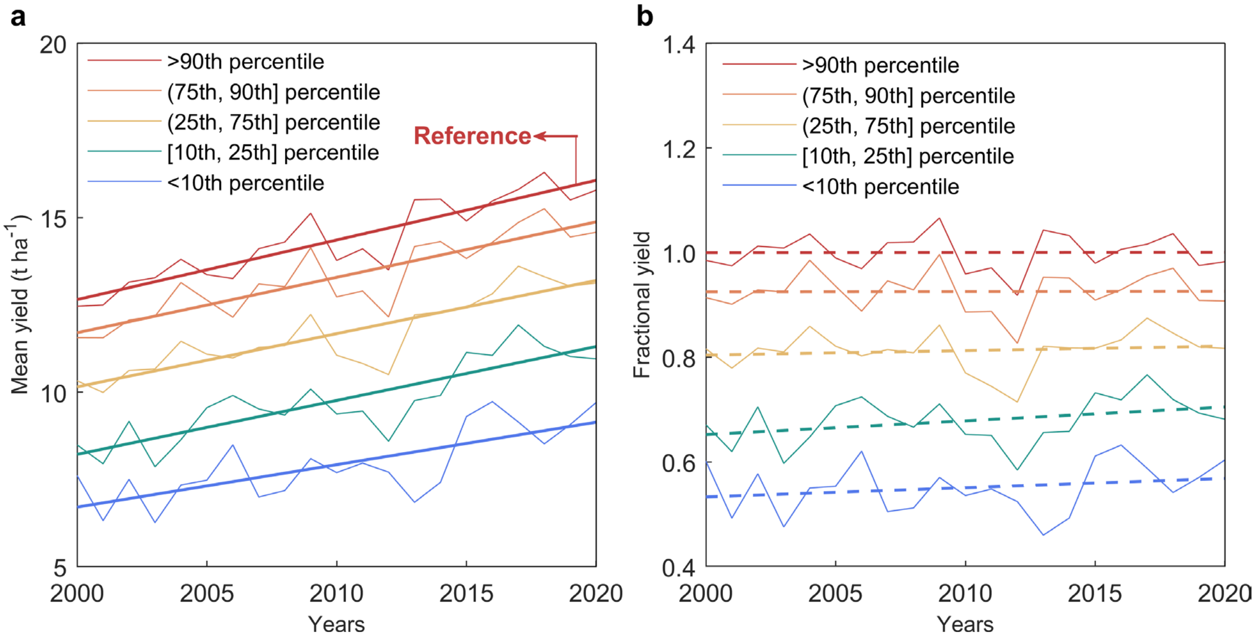

Fig. S24 | Time series of maize yield across different environmental conditions from the worst (blue line) to the best environmental conditions (red line). a, Time series of actual mean yield. The straight lines refer to trend yield derived from the least square regression. The red trend line refers to trends under the best environmental conditions, which is defined as the reference baseline. **b**, Same as panel **a** but expressed as fractional yields, calculated as the ratio of actual yield to the trend yield under the best environmental conditions. Dashed lines represent the fitted fractional yield, derived from the least square regression.

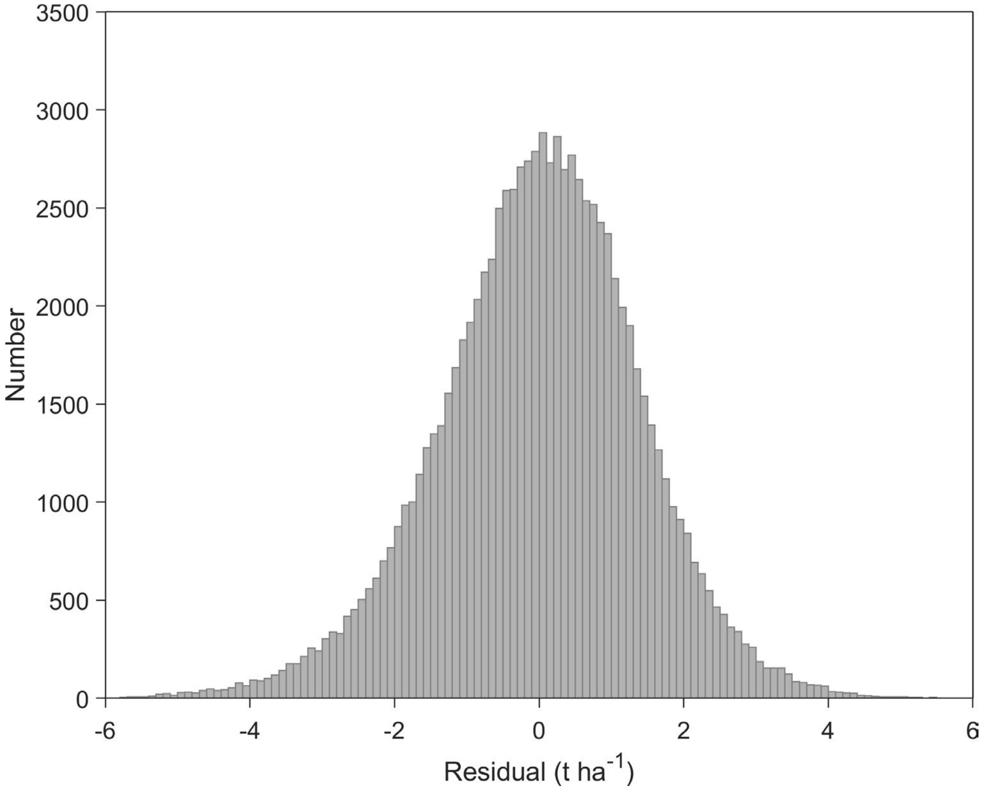

Fig. S25 | Distribution of residuals from the model in the main text (equation 7).

Step 1: Identifying the initial year of specific hybrid appeared in the field (first-trial year; *fty*)

Maize hybrids datasets (92,096 data points)

Order	Hybrids name	Planting year	Yield
1	Name 1	2000	Y_1
2	Name 2	2000	Y_2
3	Name 2	2001	Y_3
…	…	…	…
…	…	…	…
…	…	…	…
92,094	Name J	2019	$Y_{92,094}$
92,095	Name J	2020	$Y_{92,095}$
92,096	Name M	2020	$Y_{92,096}$

Identifying first-trial year

Order	First-trial year
1	2000
2	2000
3	2000
…	…
…	…
…	…
92,094	2019
92,095	2019
92,096	2020

Step 2: Extracting hybrids-specific yield data for each first-trial year

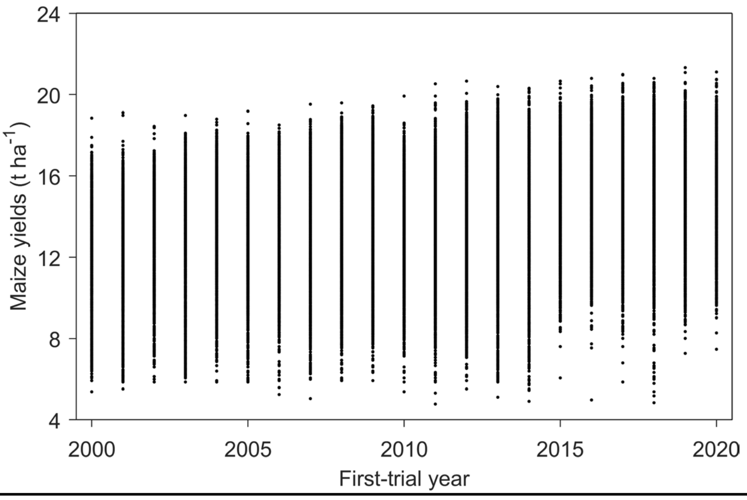

Step 3: Calculating first-trial year average yield ($\widehat{Y_{fty}}$)

$$\widehat{Y_{fty}} = \frac{\sum_{i=1}^{n} Y_{fty,i}}{n}$$

Note: *n* refers to number of hybrids for specific first-trial year; Y_{fty} is maize yield (t ha^{-1}) for specific first-trial year (*fty*).

Fig. S26 | Workflow to calculate the first trial-year average yield.

Table S1. Effect size (estimated coefficients) of weather variables on maize yield during vegetative (VEG) and grain filling periods (GFP) using the full dataset. The uncertainty is estimated based on 1,000 bootstraps.

Dependent variable: yield (t ha^{-1})	Estimated coefficients	2.5th percentile	97.5th percentile	p-value
Prcp_VEG × 100 (mm)	0.3255	0.2572	0.3896	< 0.001
$Prcp^2$_VEG × 100 (mm)	-0.0004	-0.0005	-0.0003	< 0.001
Prcp_GFP × 100 (mm)	0.9158	0.8416	0.9884	< 0.001
$Prcp^2$_GFP × 100 (mm)	-0.0016	-0.0017	-0.0014	< 0.001
VPD_b_VEG (kPa)	0.7207	0.4431	0.9928	< 0.001
VPDa_VEG (kPa)	-0.8681	-0.9585	-0.7789	0.003
VPD_b_GFP (kPa)	0.3846	0.1326	0.6526	< 0.001
VPDa_GFP (kPa)	-1.5474	-1.6147	-1.4762	< 0.001
Degrees of Freedom	92,088			
R-squared	0.63			
AIC	335,600			

Table S2. Impact of weather variables on maize yield during specific phenological periods for drought tolerant (DT) ***vs.*** non-DT hybrids. The uncertainty is estimated based on 1,000 bootstraps.

**Dependent variable: yield (t ha^{-1})**	**Estimated coefficients**	**2.5th percentile**	**97.5th percentile**
Prcp_VEG × 100mm	0.3251	0.2584	0.3914
Prcp^2_VEG × 100mm	-0.0004	-0.0005	-0.0003
Prcp_GFP × 100mm	0.9168	0.8441	0.9911
Prcp^2_GFP × 100mm	-0.0016	-0.0017	-0.0014
VPD_b_VEG (kPa)	0.7128	0.4256	0.9858
VPDb_GFP (kPa)	0.3836	0.1335	0.6246
VPDa_VEG_DT (kPa)	-0.7907	-1.0668	-0.5098
VPDa_VEG_non_DT (kPa)	-0.8691	-0.9569	-0.7749
VPDa_GFP_DT (kPa)	-1.0835	-1.4595	-0.722
VPDa_GFP_non_DT (kPa)	-1.5524	-1.6206	-1.4866
R-squared	0.63		
AIC	335,590		

Table S3. Link of public datasets for crop data and climate data

Crop datasets	Links
Iowa (2006-2020)	http://www.croptesting.iastate.edu/corn/reports.aspx
Illinois (2000-2020)	https://varietytesting.web.illinois.edu/corn/
Minnesota (2000-2020)	https://varietytrials.umn.edu/corn-grain
Ohio (2000-2020)	https://u.osu.edu/perf/archive/
Wisconsin (2000-2020)	http://corn.agronomy.wisc.edu/HT/Default.aspx
USDA-NASS (2000-2020)	https://quickstats.nass.usda.gov/
**Climate datasets**	**Links**
ISD	https://www.ncei.noaa.gov/access/metadata/landing-page/bin/iso?id=gov.noaa.ncdc:C00532
GHCNd	https://www.ncei.noaa.gov/data/global-historical-climatology-network-daily/
CMIP6	https://www.nccs.nasa.gov/services/data-collections/land-based-products/nex-gddp-cmip6

References

1. Zhao, H., *et al.* Sensitivity changes of US maize yields to extreme heat through timely precipitation patterns. *Environ. Res. Commun.* **6**, 071009 (2024).

2. Menne, M. J., Durre, I., Vose, R. S., Gleason, B. E. & Houston, T. G. An overview of the global historical climatology network-daily database. *J. Atmos. Ocean. Technol.* **29**, 897-910 (2012).

3. Fritsch, F. N. & Carlson, R. E. Monotone piecewise cubic interpolation. *SIAM J. Numer. Anal.* **17**, 238-246 (1980).

4. Sacks, W. J. & Kucharik, C. J. Crop management and phenology trends in the US Corn Belt: Impacts on yields, evapotranspiration and energy balance. *Agric. For. Meteorol.* **151**, 882-894 (2011).

5. Schlenker, W. & Roberts, M. J. Nonlinear temperature effects indicate severe damages to US crop yields under climate change. *Proc. Natl. Acad. Sci. U.S.A.* **106**, 15594-15598 (2009).

6. Akaike, H. Information theory and an extension of the maximum likelihood principle. *Selected papers of hirotugu akaike*. Springer, 1998, pp 199-213.

7. Burnham, K. P., Anderson, D. R., Burnham, K. P. & Anderson, D. R. Practical use of the information-theoretic approach. (Springer, 1998).

8. Tack, J., Barkley, A. & Nalley, L. L. Effect of warming temperatures on US wheat yields. *Proc. Natl. Acad. Sci. U.S.A.* **112**, 6931-6936 (2015).

9. Allen, R. G., Pereira, L. S., Raes, D. & Smith, M. Crop evapotranspiration-Guidelines for computing crop water requirements-FAO Irrigation and drainage paper 56. *Fao, Rome* **300**, D05109 (1998).
