# Supplementary material for: Concurrent improvements in maize yield and drought resistance through breeding advances in the U.S.Corn Belt
Source: Nat Commun. 2025 Oct 23;16:9389. doi: 10.1038/s41467-025-64454-3 (PMC12549856; doi:10.1038/s41467-025-64454-3)
Supplement: Supplementary file 3 — Reporting Summary [file 41467_2025_64454_MOESM3_ESM.pdf]

nature portfolio

Corresponding author(s): Xiaomao Lin

Last updated by author(s): Aug 26, 2025

Reporting Summary

Nature Portfolio wishes to improve the reproducibility of the work that we publish. This form provides structure for consistency and transparency in reporting. For further information on Nature Portfolio policies, see our Editorial Policies and the Editorial Policy Checklist.

Statistics

For all statistical analyses, confirm that the following items are present in the figure legend, table legend, main text, or Methods section.

n/a	Confirmed	
☐	☒	The exact sample size (*n*) for each experimental group/condition, given as a discrete number and unit of measurement
☐	☒	A statement on whether measurements were taken from distinct samples or whether the same sample was measured repeatedly
☐	☒	The statistical test(s) used AND whether they are one- or two-sided<br>*Only common tests should be described solely by name; describe more complex techniques in the Methods section.*
☐	☒	A description of all covariates tested
☐	☒	A description of any assumptions or corrections, such as tests of normality and adjustment for multiple comparisons
☐	☒	A full description of the statistical parameters including central tendency (e.g. means) or other basic estimates (e.g. regression coefficient) AND variation (e.g. standard deviation) or associated estimates of uncertainty (e.g. confidence intervals)
☐	☒	For null hypothesis testing, the test statistic (e.g. *F*, *t*, *r*) with confidence intervals, effect sizes, degrees of freedom and *P* value noted<br>*Give P values as exact values whenever suitable.*
☒	☐	For Bayesian analysis, information on the choice of priors and Markov chain Monte Carlo settings
☒	☐	For hierarchical and complex designs, identification of the appropriate level for tests and full reporting of outcomes
☒	☐	Estimates of effect sizes (e.g. Cohen's *d*, Pearson's *r*), indicating how they were calculated

Our web collection on statistics for biologists contains articles on many of the points above.

Software and code

Policy information about availability of computer code

Data collection	No custom code was used to collect data in this study.
Data analysis	Data processing, statistical analysis, and figure generation were performed using MATLAB 2023a. The code supporting main findings of this study is publicly available at https://doi.org/10.6084/m9.figshare.27038308.

For manuscripts utilizing custom algorithms or software that are central to the research but not yet described in published literature, software must be made available to editors and reviewers. We strongly encourage code deposition in a community repository (e.g. GitHub). See the Nature Portfolio guidelines for submitting code & software for further information.

Data

Policy information about availability of data

All manuscripts must include a data availability statement. This statement should provide the following information, where applicable:

- Accession codes, unique identifiers, or web links for publicly available datasets
- A description of any restrictions on data availability
- For clinical datasets or third party data, please ensure that the statement adheres to our policy

The raw maize hybrid data used in this study are publicly available at Figshare repository: https://doi.org/10.6084/m9.figshare.28960106. Historical weather data from the Integrated Surface Dataset are publicly available at https://www.ncei.noaa.gov/access/metadata/landing-page/bin/iso?id=gov.noaa.ncdc:C00532. CMIP6 data are publicly available at https://www.nccs.nasa.gov/services/data-collections/land-based-products/nex-gddp-cmip6. The source data are provided with this paper.

Research involving human participants, their data, or biological material

Policy information about studies with human participants or human data. See also policy information about sex, gender (identity/presentation), and sexual orientation and race, ethnicity and racism.

Reporting on sex and gender	N/A
Reporting on race, ethnicity, or other socially relevant groupings	N/A
Population characteristics	N/A
Recruitment	N/A
Ethics oversight	N/A

Note that full information on the approval of the study protocol must also be provided in the manuscript.

Field-specific reporting

Please select the one below that is the best fit for your research. If you are not sure, read the appropriate sections before making your selection.

☐ Life sciences ☐ Behavioural & social sciences ☒ Ecological, evolutionary & environmental sciences

For a reference copy of the document with all sections, see nature.com/documents/nr-reporting-summary-flat.pdf

Ecological, evolutionary & environmental sciences study design

All studies must disclose on these points even when the disclosure is negative.

Study description	This study analyzed a comprehensive dataset of 92,096 maize field trials conducted across the U.S. Corn Belt (2000-2020) to determine whether breeding has improved drought tolerance over time. Using the environmental index approach, we assessed breeding-driven yield trends under diverse environmental conditions. A statistical framework was employed to quantify changes in drought sensitivity during different phenological periods. Finally, we projected the impact of future drought scenarios on the yields of older and newer maize hybrids.
Research sample	Our research sample is university field performance tests of maize hybrids across five main production states, including Iowa, Illinois, Minnesota, Ohio, and Wisconsin, accounting for 50% of the US maize harvested area.
Sampling strategy	We compiled yield performance data for maize hybrids, encompassing 92,096 data points across 63 field sites from 2000 to 2020. These data included hybrid-specific yields and phenological information (planting and harvest dates). State-level maize phenological data were also extracted from the United States Department of Agriculture’s National Agricultural Statistics Service. Historical and future's climate data were derived from Integrated Surface Dataset (ISD) and CMIP6.
Data collection	All data used for the analysis is publicly available online. Therefore, no instruments or devices were used and we did not have study participants.
Timing and spatial scale	This study does not involve primary data collection.
Data exclusions	Yield performance data of maize hybrids that does not reflect reality were excluded, accounting for less than 1% of the total dataset.
Reproducibility	N/A
Randomization	N/A

Blinding	N/A

Did the study involve field work? ☐ Yes ☒ No

Reporting for specific materials, systems and methods

We require information from authors about some types of materials, experimental systems and methods used in many studies. Here, indicate whether each material, system or method listed is relevant to your study. If you are not sure if a list item applies to your research, read the appropriate section before selecting a response.

Materials & experimental systems

n/a	Involved in the study
☒	☐ Antibodies
☒	☐ Eukaryotic cell lines
☒	☐ Palaeontology and archaeology
☒	☐ Animals and other organisms
☒	☐ Clinical data
☒	☐ Dual use research of concern
☒	☐ Plants

Methods

n/a	Involved in the study
☒	☐ ChIP-seq
☒	☐ Flow cytometry
☒	☐ MRI-based neuroimaging

Plants

Seed stocks	N/A
Novel plant genotypes	N/A
Authentication	N/A
